# How Social Care Beneficiaries in Poland Rate Relative Harmfulness of Various Tobacco and Nicotine-Containing Products

**DOI:** 10.3390/ijerph14091029

**Published:** 2017-09-07

**Authors:** Marek Milcarz, Kinga Polańska, Leokadia Bak-Romaniszyn, Dorota Kaleta

**Affiliations:** 1 Department of Hygiene and Epidemiology, Medical University of Lodz, 90-647 Lodz, Poland; kinga.polanska@umed.lodz.pl (K.P.); dkaleta@op.pl (D.K.); 2 Department of Nutrition in Digestive Tract Diseases, Medical University of Lodz, 93-338 Lodz, Poland; leokadia.bak-romaniszyn@umed.lodz.pl

**Keywords:** disadvantaged groups, tobacco control, harm perception, harm reduction, public health education, cigarettes, electronic cigarettes, smokeless tobacco, water pipe

## Abstract

The aim of the study was to examine how social care beneficiaries rate the relative harmfulness of tobacco/nicotine-containing products compared to traditional cigarettes. This information is crucial for the development of effective tobacco control strategies targeting disadvantaged populations. The cross-sectional study covered 1817 respondents who were taking advantage of social aid services offered by the local social care institutions in the Piotrkowski district, via face-to-face interviews. The linear regression analysis indicated that relative to women, men consider slim cigarettes, smokeless tobacco and e-cigarettes to be more harmful than traditional cigarettes (*p* < 0.05). The smokers of traditional cigarettes reported menthol cigarettes to be less harmful than traditional cigarettes, relative to the non-smokers (*p* = 0.05). The current results demonstrate that social care beneficiaries are not aware of the fact that some products are less harmful than others. Education concerning tobacco/nicotine products should include advice on how to reduce the adverse health effects of smoking (e.g., avoiding inhalation of combusted products), while driving the awareness that no nicotine-containing products are safe.

## 1. Introduction

There is no doubt whatsoever that tobacco is the leading cause of death and disability [1,2]. While overall smoking rates among adults are slowly declining in Europe, disparities related to gender, race/ethnicity and socio-economic status (SES) persist [3]. Smoking is directly correlated with income level and education. As the level of disadvantage increases, smoking rates increase [4]. For example, 26% of adults from the United States of America (USA) who are below, and 14% of those who are at or above poverty level, smoke cigarettes [5]. Similarly, in Poland, based on the data from the Global Adult Tobacco Survey, the highest prevalence of smoking is observed among the least educated groups—smoking prevalence in 2009–2010 was 42% among those who completed education of 9 years or less versus 25% among those with over 12 years of completed education [6]. Furthermore, the following observations have been made amongst disadvantaged social groups: a younger age at commencement of smoking, a higher number of cigarettes smoked per day as well as a smaller likelihood of quitting and remaining a non-smoker [5,7,8,9]. Because of their higher tobacco consumption, lower SES smokers suffer disproportionately more from smoking-related diseases [5]. Lower-income individuals are also more likely to experience harmful consequences from exposure to environmental tobacco smoke (ETS). It should be pointed out that representatives from low SES subgroups are at a higher risk of having an additional unfavorable behavior (alcohol consumption, poor diet, lack of physical activity, etc.). In addition, they often have limited access to healthcare, defined here as a place other than traditional health services that provides antismoking activities and interventions [10,11]. Some cultural and educational barriers among disadvantaged populations can create a situation in which there are different attitudes towards tobacco smoking and its health consequences relative to the general population. As such, in order to be effective, tobacco control measures should match the perceptions and needs of these populations [9,12]. 

Low SES subgroups may be an attractive target group for the tobacco industry. As smoking rates have declined in higher-income populations, the tobacco industry has increasingly come to rely on low-income groups to sustain its consumer base, targeting this price-sensitive population through promotions and price discounting, as well as undermining policy efforts. In addition, a higher density of tobacco retailers has been found in low-income neighborhoods [5]. In order to attract new consumers and maintain existing consumers, the tobacco industry has created different types of nicotine products, with cigarettes and e-cigarettes being the most popular in Poland [13,14]. The current scientific evidence demonstrates that tobacco products, even though they differ in terms of their respective levels of harmfulness, can cause disease and death [15,16,17]. All types of cigarettes, without any exceptions, are considered to be among the most harmful tobacco products, while non-combustible products have been assessed as less harmful [18]. Bearing this in mind, slim and menthol cigarettes are not safer than regular cigarettes. On the other hand, hazards posed by smokeless tobacco products are smaller than those created by the use of combustible products. Water pipes are marketed as a safer alternative to cigarettes, but their smoke has been shown to contain substances in concentrations that are as high, or even higher, than those found in cigarette smoke. As such, they carry many of the same health risks [16,17,18,19]. Finally, e-cigarettes are considered less harmful than traditional cigarettes [20]. 

Individual perceptions and knowledge concerning negative health effects of selected forms of tobacco products may considerably influence the uptake and patterns of smoking in various socio-economic groups [18]. Studies conducted to date have shown misperceptions in the relative harmfulness of selected brands of cigarettes and cigarette substitutes in different populations [20,21,22,23,24,25]. For example, students from a rural area of Poland perceived slim cigarettes and menthol cigarettes as less harmful, which is in line with the message created by various tobacco companies. On the other hand, less popular products, such as water pipes and smokeless tobacco, were considered more harmful [26]. 

Our previous analysis indicated that the smoking prevalence among social care beneficiaries in the Piotrkowski district is much higher than that of the general population (52% of the men and 30% of the women were current smokers), with about 17% of smokers declaring willingness to quit smoking within the next month and an additional 35% within the next year [27]. Examining the harm perception patterns (beliefs about the relative harmfulness of one product compared to another) is crucial in order to develop effective measures for decreasing the burden of tobacco-related diseases and reducing tobacco use in lower-income communities. 

Although smoking rates in Poland have declined over the past decade, smoking remains prevalent among men and women. In the 2014 Multi-Centre National Population Health Examination Survey (WOBASZ), regular smoking was declared by 29.9% of men and 20.5% of women. About 75% of smokers declared their willingness to quit [28]. Despite the high prevalence of smoking in Poland and a general intention to quit, little is known about this issue, especially in relation to the assessment of how low SES adults, including beneficiaries of social and community service organizations, perceive the risks of using various tobacco and nicotine-containing products [29,30,31,32,33,34,35]. 

The aim of the study was to examine how adult social care beneficiaries rate the relative harmfulness of tobacco products, including slim and menthol cigarettes, water pipes as well as e-cigarettes and smokeless tobacco, as compared to traditional cigarettes.

## 2. Materials and Methods

### 2.1. Study Design and Population

This cross-sectional study was conducted between October 2015 and February 2016 among all adults aged 18–59 who, at the time of the study, resided in the Piotrkowski district (a socially disadvantaged rural area in the Lodzkie voivodeship—an administrative subdivision of Central Poland) and were taking advantage of aid services offered by the local social care institutions. A detailed description of the district and the methodology of the study has been published elsewhere [27,36,37,38]. The basic criterion for receiving social benefits in Poland is the level of monthly per capita income in a household. As per the relevant Social Care Act, and for the purposes of the study, socioeconomically-disadvantaged persons were defined as those who received a minimum income of no more than $158 USD (634 PLN—Polish currency) per month for a single person, and no more than $128 USD (514 PLN) per month for each family member [36,37,38].

All 3636 individuals registered in the local social care institutions who met the inclusion criteria were invited to participate, and 1817 (49.9% response rate) agreed to take part in the study. 

The study was approved by the Bioethics Committee of the Medical University in Lodz (Project Identification Code: RNN/243/15/KE), and written informed consent was obtained from all study participants.

### 2.2. Questionnaire and Study Measures

The questionnaire was adapted from the Multi-Centre National Population Health Examination Survey (WOBASZ) [29]. Face-to-face interviews were conducted by qualified interviewers at the respondents’ places of residence. 

The questionnaire covered the following socio-demographic data: age, gender, education (the number of years of completed education) as well as employment (currently employed with a permanent job, temporarily employed, disabled or retired, student, currently without a permanent or part time job, unemployed). In addition, information on cigarette use—including tobacco and e-cigarettes—was collected from the study participants. In the analysis, two categories of smoking status were created: smokers (current daily smokers—smoking one or more cigarettes per day during the past 30 days and occasional smokers—smoking less often than daily), and non-smokers.

The participants’ perceptions of the harmfulness of various tobacco and nicotine-containing products were assessed based on the methodology adapted from Smith et al. 2007 [39]. The following question was asked: “Compared to traditional cigarettes, how harmful do you think menthol, slim cigarettes, smokeless tobacco, water pipes, and e-cigarettes are?”, with the following possible answers: less harmful, just as harmful, more harmful. In the analysis, the answers received were converted into points (1 = less harmful, 2 = just as harmful, 3 = more harmful). Finally, the points were used as continuous variables to estimate the mean rating for each product [26]. 

### 2.3. Statistical Analysis 

The data supporting the study results have been provided in the (supplementary material Table S1). Taking into account the availability of the data, the analysis was performed on 1798 of 1817 respondents (99%). The results have been presented as the median and mean scores ± standard deviation (SD) for harmfulness across various products and scores, by gender and smoking status. The significance of differences between the subgroups was tested using the Mann–Whitney test. Finally, to assess the perceived harmfulness of the products, the linear regression model was applied. The variance inflation factor (VIF) was computed in order to verify the presence of multicollinearity. Regression coefficients are reported with *p* values (*p* < 0.05 was used to indicate statistical significance). The STATISTICA Windows XP version 10.0 program (StatSoft Poland Inc., Tulsa, OK, USA) was used to perform the statistical analysis.

## 3. Results

### 3.1. Characteristics of the Study Population

The characteristics of the study population have been published previously [26]. Briefly, most of the respondents were women (67%), aged 30–49 years (76%). In the studied group, only about 5% of the subjects declared more than 12 years of completed education. The population was characterized by low occupational activity, with almost 60% of the participants without a job. In the current analysis, 37.2% of the study sample declared cigarette smoking (Table 1). 

### 3.2. Perceived Relative Harm of Selected Cigarettes and Non-Cigarette Tobacco Products

Descriptive data and the mean scores for the perceived harmfulness of tobacco/nicotine products compared to traditional cigarettes are presented in Table 1, Table 2 and Table 3. E-cigarettes were scored as the least harmful form of nicotine products among those included in the study, with a mean score of 1.89 (±0.49) on a three-point scale (Table 2). The smokers of traditional cigarettes indicated a significantly lower mean score for the perceived harmfulness of slim cigarettes than the non-smokers (1.89 ± 0.43 vs. 1.96 ± 0.33; *p* = 0.03) (Table 3).

In the multivariable linear regression model, there were no statistically significant differences in the perceived relative harm of selected cigarettes and non-cigarette tobacco products relative to the age of the subjects (Table 4). The men, relative to the women, reported slim cigarettes, smokeless tobacco and e-cigarettes to be more harmful than traditional cigarettes (*p* < 0.05). The smokers of traditional cigarettes, relative to the non-smokers, reported menthol cigarettes to be less harmful (*p* = 0.05). 

## 4. Discussion

The current study indicates a high prevalence of smoking among social care beneficiaries in the Piotrkowski district. In general, all types of tobacco and nicotine-containing products were evaluated by the respondents to be as harmful as traditional cigarettes. This suggests that social care beneficiaries are not aware of the fact that some products are less harmful than others. Taking into consideration the level of exposure and low anti-tobacco activity, there is an urgent need to develop policies tailored to the needs of socially disadvantaged population groups. 

The results of our study show a higher prevalence of smoking among social care beneficiaries relative to the population of Poland as a whole [6,26]. Data from the Global Adult Tobacco Survey (GATS, 2009–2010) show that 36.8% of men and 24.4% of women are current smokers [6]. The differences are even more strongly pronounced when the studied population is compared to a rural population in the GATS study, where 35.6% of men and 20.2% of women are described as current smokers. In other countries, the proportion of smokers among social aid beneficiaries or socially disadvantaged people is also higher than that reported by the general population. For example, 53.5% of clients from social and community services in Australia report daily smoking [40]. In England, smoking is more than twice as common in the routine and manual groups than it is in the managerial and professional ones [8]. Smoking rates are also high among low income single women (46%), individuals with mental illness (41–62%), and the homeless (66–77%) [41,42,43,44,45,46,47].

According to current knowledge, cigarettes and other combustible products pose the greatest hazard to human health [18,19]. Alternative nicotine-containing products and smokeless tobacco are considered to be less harmful than cigarettes. In our study, all types of tobacco/nicotine products were scored by the respondents with a similar level of harmfulness to traditional cigarettes. In a USA national survey, 65% of respondents reported all cigarettes, regardless of type, to be more harmful than any other type of tobacco/nicotine product and more than half perceived e-cigarettes to be safer, while 9% recognized smokeless tobacco products to be safer than traditional cigarettes [16]. In our analysis, men, relative to women, rated harmfulness higher for slim cigarettes, smokeless tobacco and e-cigarettes, relative to traditional cigarettes. In addition, smokers of traditional cigarettes, relative to non-smokers, reported menthol cigarettes to be less harmful. This may indicate that parts of society receive insufficient information on the risk of particular products, which is especially relevant for rural and disadvantaged populations, which are usually poorly covered by surveillance as well as educational and promotional activities. Such a situation can be the result of a lower number of medical professionals and other resources dedicated to carrying out prevention activities, and poorer access to medical units and institutions responsible for the implementation of anti-tobacco measures. 

The impact of the tobacco industry on the perception of the harmfulness of tobacco products cannot be underestimated. All tobacco company practices—including positive image creation, recruitment strategies as well as prizes—can affect people’s understanding, attitude and perception of tobacco [48,49].

While still “unsafe”, it has been proven that smokeless tobacco products or e-cigarettes are less dangerous than traditional cigarettes [16]. It is a known fact that harm reduction beliefs surrounding these products do influence their use. Some basic principles (e.g., avoiding combustion smoke in the lungs) would likely be easy to inform the public about, while accurate physician-sourced knowledge on the relative risks of tobacco products could also be an important source of advice for smokers [16,23,50]. On the other hand, there is concern that promoting reduced-risk products (relative to traditional cigarettes) might increase the population’s intake of these “safer” substances.

What needs to be highlighted is that people with a lower SES are more likely to have lower health literacy, difficulty responding to health messages, and be more susceptible to medication myths and misperceptions, which evidently decreases the likelihood of smoking cessation [4,7]. Importantly, other factors, such as increased stress levels, competing needs, more permissive tobacco-use norms and neighborhood deprivation may play a role in successful quitting [7]. The specific knowledge deficits and beliefs that are more prevalent in disadvantaged groups could influence whether a quit attempt is made, and whether the attempt is made using an evidence-based method. Beliefs that cessation medications are ineffective, dangerous, addictive or too costly are more prevalent among those living in poverty, and correlate negatively with the intention to quit and quitting attempts. Continued tobacco-control progress requires addressing these specific populations. Efforts to address and modify their beliefs might result in more attempts to quit using evidenced-based methods.

The current study has several strengths. Firstly, for the first time, harm perceptions of selected cigarettes and non-cigarette tobacco products have been collected from a socially disadvantaged population of social care beneficiaries from a rural district in Poland. Secondly, from all those invited to participate in the study, about half agreed to take part in the survey. As such, the participation rate is comparable to that obtained in other surveys in Poland (GATS Poland: 60%, WOBASZ II: 45%) [6,29]. Finally, the interviewer-administered questionnaires completed during the face-to-face interview produced higher values of sensitivity and specificity than the self-administered questionnaires, and helped to reduce the non-response rate [51]. 

Nevertheless, the limitations of the study should also be mentioned. All the estimates in our assessment were based on self-reports, which might be affected by reporting bias. The social care beneficiaries might have also under- or over-reported their assessment of the harmfulness of selected tobacco products. The assessment of the harmfulness of different tobacco and nicotine-containing products relative to traditional cigarettes (rather than rating each product) may also pose some limitations to the study. The cross-sectional design is also identified as an additional study limitation. This type of study tends to make observations at a single point in time, which makes it impossible to observe changes in the perception of harmfulness over longer periods of time. This is particularly crucial in the context of newer tobacco product formulations, where public awareness, knowledge and risk perception are likely to be dynamic [16]. Unfortunately, the findings of the study do not allow us to state that the improvement of living conditions in vulnerable groups will provide them with better skills to protect their health. 

## 5. Conclusions

The current study indicates a high prevalence of smoking among social care beneficiaries in the Piotrkowski district and underlines their misperceptions of the relative harmfulness of selected brands of cigarettes and cigarette substitutes. The findings strongly suggest a need for anti-tobacco activities dedicated to this group, with simultaneous accurate and effective information about the relative harm of different tobacco products. Education concerning tobacco/nicotine products should include advice on how to reduce the adverse health effects of smoking (e.g., avoiding inhalation of combusted products), and at the same time make people aware that no tobacco product is safe. 

## Figures and Tables

**Table 1:** Perceived harmfulness of tobacco/nicotine products.

Harmfulness of Selected Products		Menthol Cigarettes			Slim Cigarettes			Smokeless Tobacco			Water Pipe			E-Cigarettes		
		Less Harmful	As Harmful	More Harmful	Less Harmful	As Harmful	More Harmful	Less Harmful	As Harmful	More Harmful	Less Harmful	As Harmful	More Harmful	Less Harmful	As Harmful	More Harmful
Overall N = 1798	N	218	1477	103	186	1541	71	233	1458	107	220	1464	114	326	1340	132
	%	12.1	82.1	5.7	10.3	85.7	4.0	13.0	81.1	5.9	12.2	81.4	6.3	18.1	74.5	7.3
	95% CI	10.6–13.6	80.3–83.9	4.6–6.8	8.9–11.7	84.1–87.3	3.4–4.9	11.4–14.6	79.3–82.9	4.8–7.0	10.7–13.7	79.6–83.2	5.2–7.4	16.3–19.9	72.5–76.5	6.1–8.5
Smoker N = 637	N	80	514	43	96	513	28	100	485	52	87	502	48	127	448	62
	%	12.6	80.7	6.7	15.1	80.5	4.4	15.7	76.1	8.2	13.7	78.8	7.5	19.9	70.3	9.7
	95% CI	10.0–15.2	77.6–83.8	4.8–8.6	12.3–17.9	77.4–83.6	2.8–6.0	12.9–18.5	72.8–79.4	6.1–10.3	11.0–16.4	75.6–82.0	5.4–9.6	46.0–53.8	66.7–73.9	7.4–12.0
E-cigarettes (user or dual user) N = 42	N	7	30	5	4	33	5	5	30	7	4	29	9	16	20	6
	%	16.7	71.4	11.9	9.5	78.6	11.9	11.9	71.4	16.7	9.5	69.0	21.4	38.1	47.6	14.3
	95% CI	5.4–28.0	57.7–85.1	2.1–21.7	0.6–18.4	66.2–91.0	2.1–21.7	2.1–21.7	57.7–85.1	5.4–28.0	0.6–18.4	55.0–83.0	9.0–33.8	23.4–52.8	32.5–62.7	3.7–24.9
Non-Smoker N = 1119	N	131	933	55	86	995	38	128	943	48	129	933	57	183	872	64
	%	11.7	83.4	4.9	7.7	88.9	3.4	11.4	84.3	4.3	11.5	83.4	5.1	16.4	77.9	5.7
	95% CI	9.8–13.6	81.2–85.6	3.6–6.2	6.1–9.3	87.1–90.7	2.3–4.5	9.5–13.3	82.2–86.4	3.1–5.5	9.6–13.4	81.2–85.6	3.8–6.4	14.2–18.6	75.5–80.3	4.3–7.1

**Table 2:** Median and mean scores for the perceived harmfulness of selected tobacco/nicotine products by gender.

Product *	Overall	Males	Females	Z (*p* Value) ^^^
	Median			
	Mean ± SD			
Menthol Cigarettes	2.00	2.00	2.00	−0.14 (0.22)
	1.94 ± 0.42	1.93 ± 0.44	1.94 ± 0.41	
Slim Cigarettes	2.00	2.00	2.00	−0.07 (0.94)
	1.94 ± 0.37	1.94 ± 0.40	1.94 ± 0.36	
Smokeless Tobacco	2.00	2.00	2.00	−0.59 (0.56)
	1.93 ± 0.43	1.92 ± 0.46	1.94 ± 0.41	
Water Pipe	2.00	2.00	2.00	0.02 (0.98)
	1.94 ± 0.43	1.94 ± 0.45	1.94 ± 0.42	
E-cigarettes	2.00	2.00	2.00	−0.90 (0.30)
	1.89 ± 0.49	1.87 ± 0.52	1.90 ± 0.48	

Note: * Scoring has been given to each product based on its harmfulness compared to traditional cigarettes with 1 = less harmful, 2 = as harmful, 3 = more harmful (the points were used as continuous variables to estimate the mean rating for each product). SD, standard deviation. **^^^** Z-statistics in the Mann–Whitney test.

**Table 3:** Median and mean scores for the perceived harmfulness of selected tobacco/nicotine products by cigarette smoking status.

Product *	Smoker	Non-Smoker	Z (*p* Value) ^^^
	Median		
	Mean ± SD		
Menthol Cigarettes	2.00	2.00	0.28 (0.78)
	1.94 ± 0.44	1.93 ± 0.40	
Slim Cigarettes	2.00	2.00	**−2.17 (0.03)**
	1.89 ± 0.43	1.96 ± 0.33	
Smokeless Tobacco	2.00	2.00	−0.23 (0.82)
	1.93 ± 0.48	1.93 ± 0.39	
Water Pipe	2.00	2.00	0.05 (0.96)
	1.94 ± 0.46	1.94 ± 0.40	
E-cigarettes	2.00	2.00	−0.007 (0.99)
	1.90 ± 0.54	1.89 ± 0.46	

Note: * Scoring has been given to each product based on its harmfulness compared to traditional cigarettes with 1 = less harmful, 2 = as harmful, 3 = more harmful (the points were used as continuous variables to estimate the mean rating for each product). SD, standard deviation. **^^^** Z-statistics in the Mann–Whitney test. Regression coefficients significantly different from 0 are marked in bold.

**Table 4:** Associations between the perceived harmfulness of selected tobacco/nicotine products and age, gender and cigarette smoking status.

Variables	Perceived Harmfulness of Selected Products *				
	Menthol Cigarettes	Slim Cigarettes	Smokeless Tobacco	Water Pipe	E-Cigarettes
	β (*p* Value)				
Age (continuous variable)	0.29 (0.09)	−0.09 (0.60)	−0.27 (0.10)	−0.29 (0.11)	−0.21 (0.17)
Gender (male vs. female)	0.01 (0.54)	**0.42 (0.03)**	**0.41 (0.02)**	0.11 (0.58)	**0.45 (0.01)**
Smoking Status (smoker vs non-smoker)	**−0.349 (0.05)**	0.071 (0.69)	0.223 (0.18)	−0.105 (0.57)	0.293 (0.07)

Note: * Scoring has been given to each product based on its harmfulness compared to traditional cigarettes with 1 = less harmful, 2 = as harmful, 3 = more harmful (the points were used as continuous variables to estimate the mean rating for each product). SD, standard deviation. The regression coefficients (β) presented for each variable reflect their association with perceived harmfulness after adjustment for confounding from the other factors. Regression coefficients significantly different from 0 are marked in bold.

## Supplementary Materials

The following are available online at www.mdpi.com/1660-4601/14/9/1029/s1, Table S1: Data set.
